# Anti-Bacterial and Anti-Fungal Properties of a Set of Transition Metal Complexes Bearing a Pyridine Moiety and [B(C_6_F_5_)_4_]_2_ as a Counter Anion

**DOI:** 10.3390/molecules30153121

**Published:** 2025-07-25

**Authors:** Ahmed K. Hijazi, Mohammad El-Khateeb, Ziyad A. Taha, Mohammed I. Alomari, Noor M. Khwaileh, Abbas I. Alakhras, Waleed M. Al-Momani, Ali Elrashidi, Ahmad S. Barham

**Affiliations:** 1 Department of Chemistry, College of Arts and Sciences, University of Petra, P.O. Box 961343, Amman 11196, Jordan; mohammed.alomari@uop.edu.jo; 2 Department of Chemical Sciences, Faculty of Science and Arts, Jordan University of Science and Technology, P.O. Box 3030, Irbid 22110, Jordan; kateeb@just.edu.jo (M.E.-K.); tahaz33@just.edu.jo (Z.A.T.); noorkhwaileh@sci.just.edu.jo (N.M.K.); 3 Department of Chemistry, College of Science, Imam Mohammad Ibn Saud Islamic University, P.O. Box 5701, Riyadh 11432, Saudi Arabia; aakhrasi@imam.edu.sa; 4 Department of Basic Medical Sciences, Faculty of Medicine, Yarmouk University, Irbid 21163, Jordan; waleed.momani@yu.edu.jo; 5 Electrical Engineering Department, College of Engineering, University of Business and Technology, Jeddah 23435, Saudi Arabia; a.elrashidi@ubt.edu.sa; 6 Engineering Mathematics Department, Faculty of Engineering, Alexandria University, Alexandria 21544, Egypt; 7 Department of Chemistry, School of Science, The University of Jordan, Amman 11942, Jordan; a.barham@ju.edu.jo

**Keywords:** metal(II) complexes, antimicrobial, MIC, counter anion, pyridine

## Abstract

Background: Transition metal complexes incorporating fluorinated counter anions represent a significant class of compounds with broad applications in industry, pharmaceuticals, and biomedicine. These fluorinated anions are known to enhance the solubility, stability, and reactivity of the complexes, thereby expanding their functional utility in various chemical and biological contexts. Methods: A set of metal(II) complexes of the general formula [MPy_6_][B(C_6_F_5_)_4_]_2_ where (Py = pyridine, M = Mn (**1**), Fe (**2**), Co (**3**), Ni (**4**), Cu (**5**), Zn (**6**)) have been synthesized by direct reaction of metal halides and pyridine in the presence of Ag[B(C_6_F_5_)_4_]. The complexes were characterized using different techniques to assure their purity, such as elemental analysis (EA), electron paramagnetic resonance (EPR) spectroscopy, thermogravimetric analysis (TGA), ultraviolet–visible (UV–Vis) spectroscopy, ^11^B-NMR, ^1^H-NMR, and FT-IR spectroscopy. The antimicrobial and antifungal properties against different types of bacteria and fungi were studied for all prepared complexes. Results: The synthesized complexes exhibited broad-spectrum antimicrobial activity, demonstrating variable efficacy compared to the reference antibiotic, oxytetracycline (positive control). Notably, complex **6** displayed exceptional antibacterial activity against *Streptococcus pyogenes*, with a minimum inhibitory concentration (MIC) of 4 µg/mL, outperforming the control (MIC = 8 µg/mL). Complexes **1**, **2**, and **4** showed promising activity against *Shigella flexneri*, *Klebsiella pneumoniae*, and *Streptococcus pyogenes*, each with MIC values of 8 µg/mL. Conversely, the lowest activity (MIC = 512 µg/mL) was observed for complexes **3**, **5**, and **6** against *Pseudomonas aeruginosa*, *Escherichia coli*, and *Klebsiella pneumoniae*, respectively. Regarding antifungal properties, complexes **5** and **6** demonstrated the highest activity against *Candida albicans*, with MIC values of 8 µg/mL, equivalent to that of the positive control, fluconazole. Density functional theory (DFT) calculations confirmed an overall octahedral coordination geometry for all complexes, with tetragonal distortions identified in complexes **3**, **4**, and **5**.

## 1. Introduction

Over the past few decades, the development of bacterial resistance to currently available antibiotics has grown to be a serious worldwide health concern. As a result, finding and creating novel antibacterial agents continues to be a top focus. Coordination of metal ions with physiologically active ligands is a promising tactic that is known to improve the pharmacological characteristics of both substances. These metal-based complexes provide a flexible framework for creating new antibacterial substances that are more effective against resistant bacteria [1,2,3,4,5,6].

Metal organonitrile and organoamine complexes have diverse applications in chemistry as catalysts and as promising materials [7,8,9,10,11,12]. Pyridine derivatives and their substituted analogs are well known for their diverse biological activities, including antibacterial [13], antifungal [14], antimicrobial [15,16], and anti-lung cancer properties [17]. It has been well established that the biological activity of certain organic compounds can be enhanced through interaction with metal ions during metabolic processes, underscoring the critical role of inorganic species in various biochemical pathways [18,19]. Coordination complexes of transition metals, in particular, have been widely reported for their antimicrobial properties [20,21,22].

The presence of non- or weakly-coordinated counter anions in organometallic complexes has a very important role in enhancing the reactivity of these complexes [23,24,25]. Complexes having a general formula of [M(NCR)_n_][FA]_m_ (where M: 1st row transition metal; R: C_2_H_5_, C_6_H_5_; FA: fluorinated counter anion) have been known for their role in many organic reactions as initiators or precursors for cyclopropanation [26,27], polymerizations [28,29,30,31,32,33,34], and aziridination reactions [35,36]. These kinds of anions have a great effect on stabilizing the cationic part of the complexes, as well as the metal accessibility for substrate coordination in intermediate species. In addition, complexes containing fluoride have been reported in the treatment of many diseases and are applied as pharmaceuticals [37]. Due to its high electronegativity as well as its high binding affinity, the presence of fluorine atom in the system might enhance the metabolic stability, and at the same time alter the physio-chemical properties [38].

The biological significance of metal complexes having fluorinated anions has been extensively studied, especially in light of their antioxidant and antibacterial properties. Recent research has concentrated on transition metal complexes in different oxidation states (+1, +2, +3), using a variety of ligand structures and anions, including fluorinated and non-fluorinated species. These efforts aim to highlight the potential of such structurally simple complexes as promising candidates in antimicrobial drug discovery and pharmaceutical development [39,40,41,42,43].

In continuation to our research work and interest in solvent-ligated organometallic complexes bearing a variety of fluorinated and non-fluorinated counter anions, herein we report the synthesis, characterization of transition metal complexes bearing a pyridine moiety, and B(C_6_F_5_)_4_ as a counter anion with the general formula [M(Py)_6_][B(C_6_F_5_)_4_]_2_ where (M = Mn, Fe, Co, Ni, Cu, Zn). The antibacterial properties of these complexes against a variety of Gram-positive and Gram-negative bacterial strains and one fungi strain are reported.

## 2. Results and Discussion

The preparation of K[B(C_6_F_5_)_4_] was carried out by reacting pentafluorophenyl bromide with n-butyl lithium in the presence of potassium chloride forming the potassium salt K[B(C_6_F_5_)_4_] (Equations (1)–(3)). The reaction of potassium salt with silver nitrate produced the corresponding silver salt (Equation (4)) which was reacted with metal(II) halides in acetonitrile to yield [M(CH_3_CN)_6_][B(C_6_F_5_)_4_]_2_ (Equation (5)). The complexes [MnPy_6_][B(C_6_F_5_)_4_]_2_ (**1**), [FePy_6_][B(C_6_F_5_)_4_]_2_ (**2**), [CoPy_6_][B(C_6_F_5_)_4_]_2_ (**3**), [NiPy_6_][B(C_6_F_5_)_4_]_2_ (**4**), [CuPy_6_][B(C_6_F_5_)_4_]_2_ (**5**), and [ZnPy_6_][B(C_6_F_5_)_4_]_2_ (**6**) were synthesized by adding an excess amount of dry pyridine to a solution of acetonitrile complexes in CH_2_Cl_2_ (Equation (6)).

(1) $$C_{6}F_{5}Br+n\text{-}BuLi\overset{Et_{2}O\;}{\underset{-78\;{}^{\circ}C}{\to }}C_{6}F_{5}Li+n\text{-}BuBr$$

(2) $${4C}_{6}F_{5}Li+BCl_{3}\overset{Et_{2}O\;}{\underset{-78\;{}^{\circ}C}{\to }}Li\left[B{\left(C_{6}F_{5}\right)}_{4}\right]+3LiCl$$

(3) $$Li\left[B(C_{6}{F_{5})}_{4}\right]+KCl\overset{R.T.}{\underset{{Et}_{2}O/H_{2}O}{\to }}K\left[B(C_{6}{F_{5})}_{4}\right]+LiCl$$

(4) $$K\left[B(C_{6}{F_{5})}_{4}\right]+AgNO_{3}\overset{CH_{3}CN}{\underset{R.T./{Et}_{2}O}{\to }}Ag[B(C_{6}{F_{5})}_{4}]+KNO_{3}$$

(5) $$2Ag[B\left(C_{6}{F_{5})}_{4}\right]+MCl_{2\left(s\right)}\overset{CH_{3}CN}{\underset{R.T.}{\to }}[M(CH_{3}CN{)}_{6}][B(C_{6}{F_{5})}_{4}{]}_{2}+2AgCl$$

(6) $$[M(CH_{3}CN{)}_{6}][B(C_{6}{F_{5})}_{4}{]}_{2}\overset{CH_{2}Cl_{2}}{\underset{NC_{5}H_{5}}{\to }}[M(NC_{5}H_{5}{)}_{6}][B(C_{6}F_{5}{)}_{4}{]}_{2}$$

where M = Mn, Fe, Co, Ni, Cu, Zn.

### 2.1. FT-IR Spectroscopy

**Figure 1:**
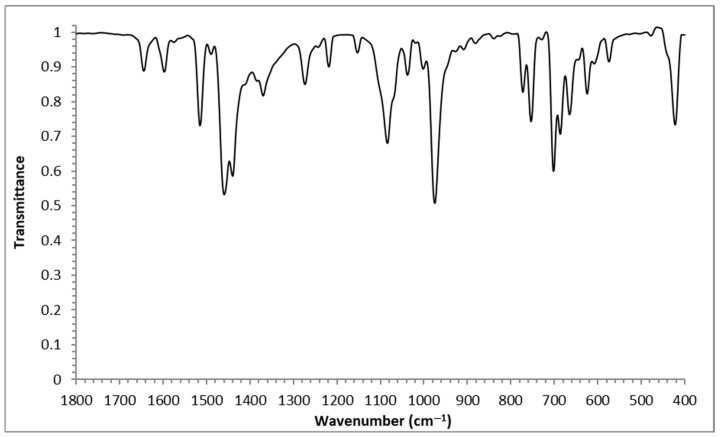
FT-IR spectrum of complex **5**.

The infrared (IR) spectral data of all complexes are listed in Table 1. Figure 1 and Figures S1–S5, show the IR spectra for all complexes. Complexes **1**–**6** exhibit one sharp ν(C=N) absorptions in the range of 1642–1644 cm^−1^ and one sharp ν(C=C) absorptions at 1597 and 1596 cm^−1^. In the range of 422–420 cm^−1^, **1**–**6** show one sharp absorptions for ν(M-N). The spectra also contain peaks in the range of 621–662 cm^−1^ assigned to the stretching bands of the M–N bond. These results indicate that pyridine is coordinated with metal(II) complexes [44].

The observed IR-active modes in the context of DFT-optimized structures are performed and discussed (Figures S17–S22 and Table S1). The calculated IR spectrum of **6** exhibits a sharp, intense peak at 640 cm^−1^ (intensity ~200), assigned to the symmetric Zn–N stretching vibration, which reflects the regular octahedral geometry of the d^10^ Zn(II) center, where all six Zn–N bonds are equivalent (2.29 Å). The absence of peak splitting or broadening further confirms the absence of Jahn–Teller distortion, consistent with its closed-shell electronic configuration. In contrast, that of **5** displays multiple M–N stretching peaks at 626–654 cm^−1^, with lower intensities (~50–100), indicative of Jahn–Teller distortion exhibiting an axial elongation (2.62 Å vs. 2.09–2.10 Å equatorial), leading to weaker axial bonds and a broader vibrational profile. Complex **4** shows an equatorial similar trend, with peaks at 622–670 cm^−1^ and significant axial elongation (3.36 Å vs. 1.93 Å), attributed to steric crowding and limited π-backdonation due to Ni(II)’s smaller ionic radius (68 pm). For **3**, with a d^7^ configuration, it exhibits Jahn–Teller distortion, evidenced by peaks at 625–655 cm^−1^ and axial bond elongation (2.55 Å vs. 2.03 Å equatorial). However, for the high-spin complexes (**1** and **2**)**,** intermediate M–N stretching frequencies (643–646 cm^−1^, 630–651 cm^−1^), with relatively symmetric peak profiles, reflect their weaker distortion tendencies.

Higher-frequency vibrations of the pyridine ring predominate in the 1000–1100 cm^−1^ range. At 1043 cm^−1^ (intensity ~550), complex **6** has an extraordinarily strong peak that suggests rigid, symmetric ligand coordination. Broader peaks in this range (1032–1055 cm^−1^) are seen in complexes **4** and **5**, which may indicate ligand distortion brought on by asymmetric metal environments. The remaining complexes show similar patterns, albeit with lower intensities, which correspond to their different levels of geometric distortion. These infrared results are in perfect agreement with the experimentally reported results.

### 2.2. ^11^B and ^1^H –NMR Spectral Data

**Figure 2:**
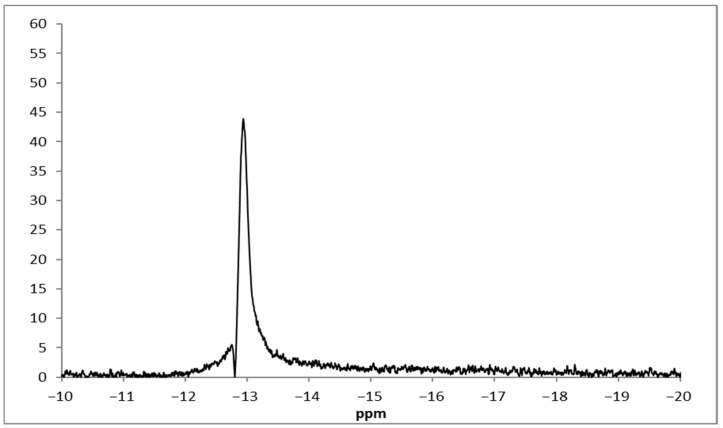
^11^B-NMR of complex **4**.

For complexes **1**–**6**, the ^11^B-NMR spectra were recorded in deuterated dimethyl sulfoxide (DMSO-*d*_6_), in the range −20 to −10 ppm. In general, the chemical shifts for ^11^B are found between −60 and 90 ppm and depend on the complexes’ strengths. Moreover, the ^11^B shifts for M^+^BR_4_^−^ systems were found to be between −31 and −6 ppm [45,46,47]. For complexes **1**–**6**, a singlet peak is observed in the range of −11.987 to −12.869 ppm as presented in Figure 2 and Figures S6–S10 which are assigned to boron of the anion. These results are similar to previously reported ones [41,42,43]. The ^1^H-NMR spectrum of complex **6** (DMSO-*d*_6_) shows the pyridine protons as doublets and triplets in the aromatic region (Figure S11) and are similar to those reported for similar systems [48,49].

### 2.3. Elemental Analysis of Complexes **1**–**6**

**Figure 3:**
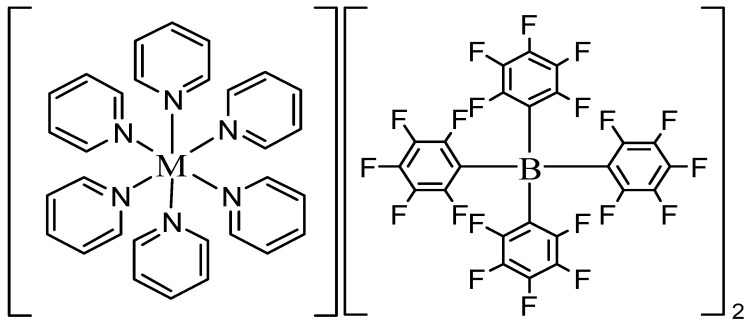
Proposed structure of all complexes (M = Mn, Fe, Co, Ni, Cu, Zn).

Elemental analysis and synthetic yields for complexes **1**–**6** are summarized in Table 2. The experimentally determined elemental compositions (C, H, N, and F) are in good agreement with the calculated values based on the proposed molecular formula (Figure 3), thereby supporting the accuracy of the assigned molecular compositions.

### 2.4. Electron Paramagnetic Resonance (EPR) Spectra of **1**, **2**, and **5**

**Figure 4:**
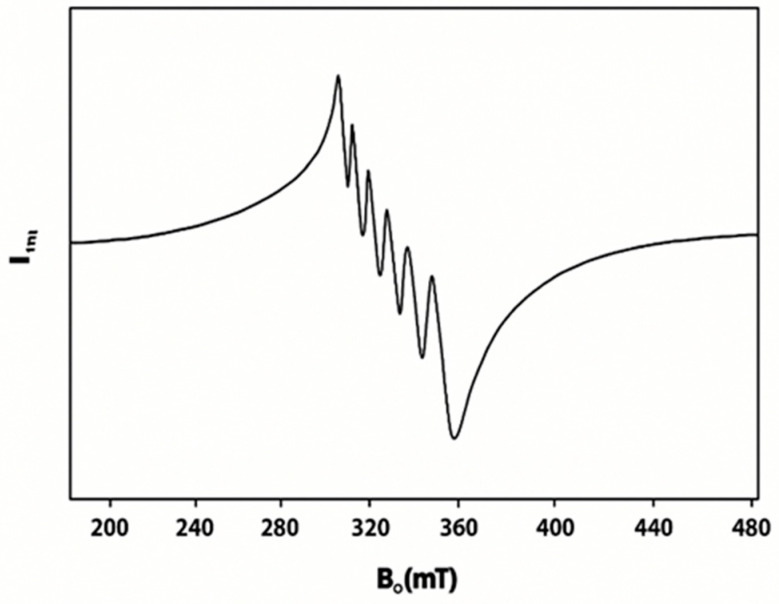
X-band EPR spectrum of complex **1**.

**Figure 5:**
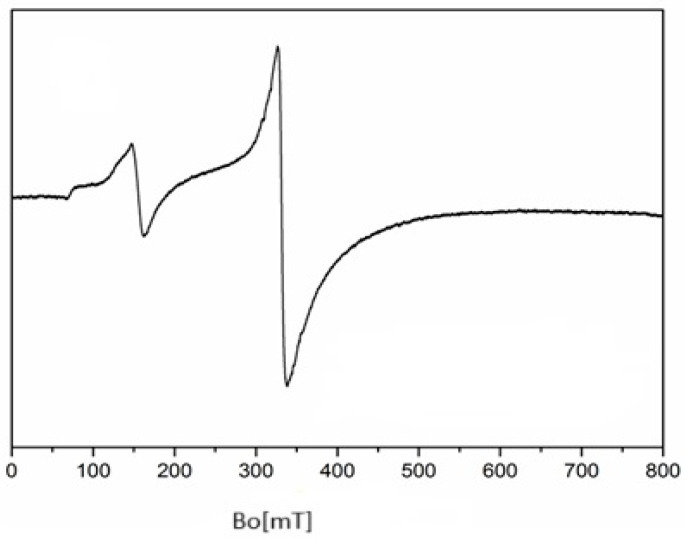
X-band EPR spectrum of complex **2**.

The EPR spectrum of complex **1** exhibits g-values of (g_iso_ = 2.001; g_┴_ = 2.000; g_‖_ = 2.002) (Figure 4), which consistent with those typically observed for octahedral Mn^2+^ complexes and are close to the free electron value (g_e_ = 2.0023). These values indicate minimal spin–orbit coupling and support the proposed geometry. Moreover, the hyperfine coupling constants show good agreement with previously reported Mn^2+^ complexes, further validating the structural assignment [28,50,51]. Moreover, for complex **2** (Figure 5), the found g values (g_┴_ = 1.992; g_‖_ = 1.998) are comparable to previously reported values of Fe^2+^ ions [52].

**Figure 6:**
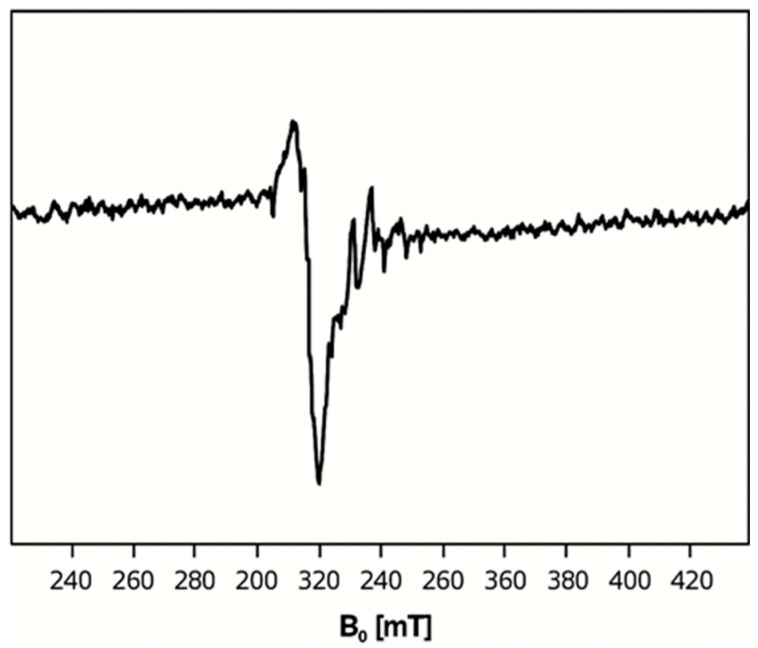
X-band EPR spectrum of complex **5**.

Complex **5** has g-values (g_iso_ = 2.174; g_┴_ = 2.083; g_‖_ = 2.296) (Figure 6) comparable to those found in previously reported copper complexes [53,54]. Since g_‖_ value is higher than g_┴_, this suggests that either the Cu ion has a normal *O_h_* coordination or distorted through elongation in the z^2^ axis.

### 2.5. Thermal Gravimetric Analysis (TGA) of Complexes **1**–**6**

Thermogravimetric (TG) and differential thermogravimetric (DTG) analysis for complexes were carried out within a temperature range from 30 °C up to 800 °C under N_2_ flow. The thermal analysis data of the complexes **1**–**6** are listed in Table 3.

**Figure 7:**
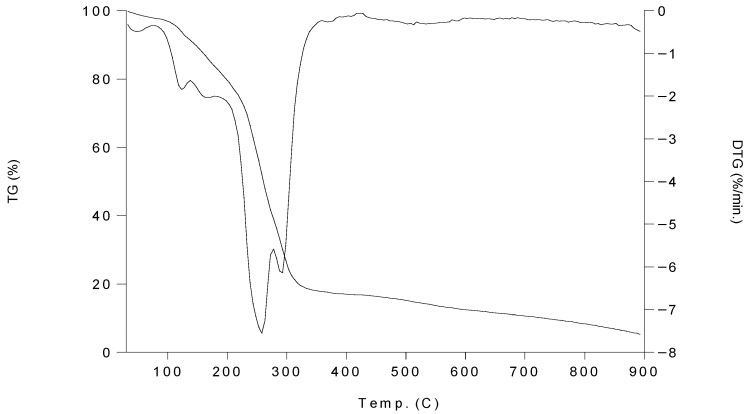
TGA/DTG curves of complex **1**.

As a representative example, the TGA/DTG curve of complex **1** shows that the first decomposition temperature range is 94–196 °C, being associated with a mass loss of 16.88 wt.%. The first decomposition step corresponds to the loss of four pyridine ligands while the loss in the second decomposition range (196–281 °C) is associated with a mass loss of 43.10 wt.% and corresponds to the loss of the two remaining pyridine ligands with anion fragmentation (Figure 7). The TGA/DTG curves of complexes **2**–**6** are represented through Figures S12–S16, respectively. The residual mass in all complexes indicates the presence of metals fluoride (M^II^F_2_) generated from anion fragments and reaction with the metal.

### 2.6. Ultraviolet–Visible (UV–Vis) Spectra of Complexes **1**–**6**

UV–Vis absorption spectra of the complexes were recorded in ethyl acetate at room temperature. The absorption maxima along with their corresponding absorbance values are listed in Table 4. The multiple bands observed at shorter wavelengths in each spectrum are assigned to π→π^*^ transitions of the ligand framework, while the band at the longest wavelength is attributed to an n→π^*^ transition.

### 2.7. Structural Analysis of Complexes **1**–**6**

**Figure 8:**
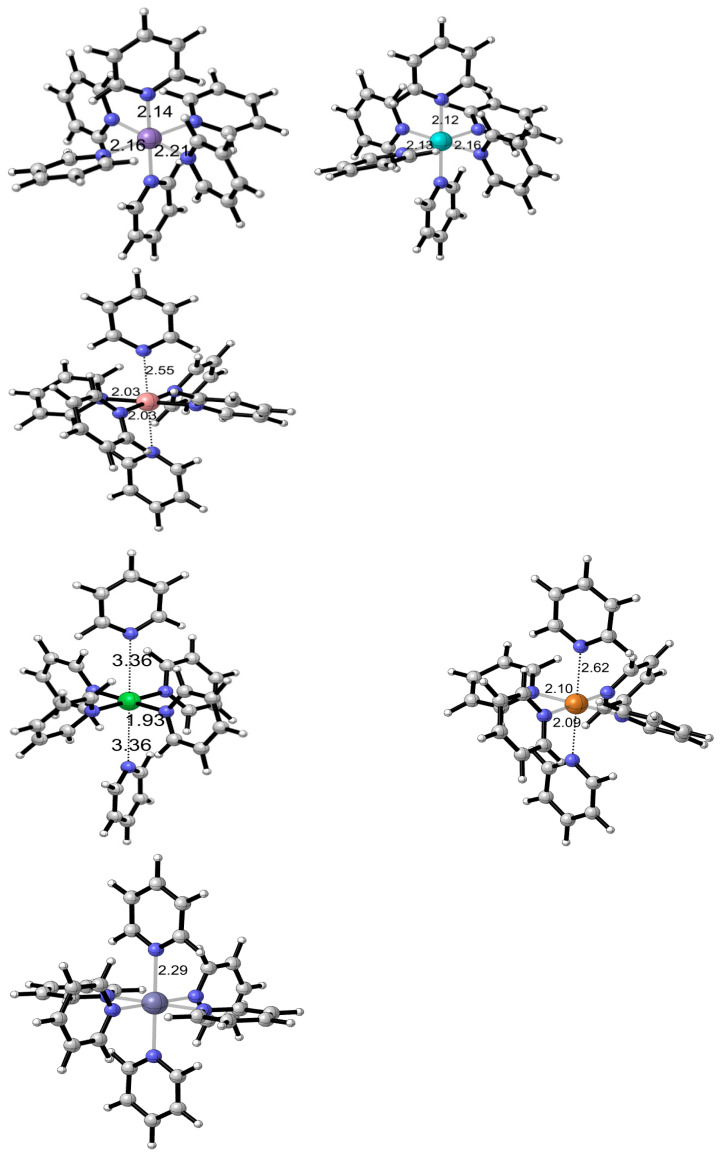
Optimized molecular geometries of [MPy_6_]^2+^ complexes obtained from DFT calculations using the ωB97XD functional, with LANL2DZ pseudopotential basis set for metal atoms and 6-31+G(d) basis set for non-metal atoms. Metal–nitrogen (M–N) bond distances are shown in angstroms (Å).

The systematic structural analysis of first-row transition metal complexes with pyridine ligands, [MPy_6_]^2+^ where M = Mn, Fe, Co, Ni, Cu, and Zn, reveals interesting trends in their molecular geometries as determined through DFT calculations, as shown in Figure 8.

Three of these complexes exhibit regular octahedral geometries, with [MnPy_6_]^2+^, [FePy_6_]^2+^, and [ZnPy_6_]^2+^ showing relatively uniform metal–nitrogen bond distances across all six coordinated pyridine ligands. This structural regularity reflects the electronic configurations of these metal centers and their interaction with the π-accepting pyridine ligands. A progressive decrease in M–N bond lengths is observed traversing from Mn to Ni, consistent with the decrease in cationic radii across the first-row transition series due to increased effective nuclear charge.

The [CoPy_6_]^2+^ and [CuPy_6_]^2+^ complexes present a notable deviation from regular octahedral geometry, displaying a characteristic Jahn–Teller distortion [55,56]. This distortion manifests as an elongation of the two *trans* axial M–N bonds relative to the four equatorial M–N bonds, a phenomenon attributed to the d^7^ and d^9^ electronic configuration of Co(II) and Cu(II), respectively, which results in asymmetric occupation of the e_g_ orbitals. This structural distortion serves to remove the electronic degeneracy and lower the overall energy of the complex.

The axial elongation observed in [NiPy_6_]^2+^ is affected by both ligand-field and steric influences. Pyridine acts as a π-accepting ligand, enabling back-donation from the filled *d* orbitals of the metal to its π^*^ antibonding orbitals, which in turn affects the strength of the metal–ligand bonds. However, the relatively lower energy of Ni(II)’s d orbitals, in comparison to earlier transition metals such as Mn or Fe, may limit the degree of π-back-donation, thereby slightly weakening the axial Ni–N bonds. Moreover, the steric hindrance caused by the six bulky pyridine ligands could exacerbate the axial elongation. This effect is particularly pronounced because Ni(II) is the smallest ion among the six, with a radius of 68 pm, in contrast to the larger radii of 83 pm for Mn(II), 78 pm for Fe(II), 75 pm for Co(II), 73 pm for Cu(II), and 74 pm for Zn(II) [57]. The crowded coordination environment promotes this distortion to minimize interactions between the ligands.

### 2.8. Antimicrobial Activity of Complexes **1**–**6**

#### 2.8.1. Agar Well Diffusion

Some of the synthesized complexes exhibit notable zones of inhibition, according to a comparative analysis of zones of inhibition seen for all complexes using the agar well diffusion method against human pathogenic bacteria (Table 5).

#### 2.8.2. Determination of Minimum Inhibitory Concentration

The MIC of the complexes against different types of Gram-positive and Gram-negative bacteria, *Escherichia coli* (*Ec*); *Proteus mirabilis* (*Pm*); *Shigella flexneri* (*Sf*); *Klebsiella pneumoniae* (*Kp*); *Pseudomonas aeruginosa* (*Pa*); *Citrobacter freundii* (*Cf*) (as Gram-negative) and *Staphylococcus aureus* (*Sa*); *Streptococcus pyogenes* (*Sp*) (as Gram-positive), and *Candida albicans* (*Ca*) as fungi, were determined and tabulated (Table 6).

Complex **6** showed an extraordinary antibacterial activity against (*streptococcus pyogenes*) with MIC value of 4 µg/mL compared to the value of 8 µg/mL for the +ve control. Complexes **1**, **2**, and **4** showed excellent promising activities against *Shigella flexneri*, *Klebsiella pneumoniae*, and *Streptococcus pyogenes*, with MIC values of 8 µg/mL, respectively. The lowest activities with values of 512 µg/mL have been observed for complexes **3**, **5**, and **6** against *Pseudomonas aeruginosa*, *Escherichia coli*, and *Klebsiella pneumoniae*, respectively. Complexes **5** and **6** showed the highest antifungal activities against *Candida albicans* with MIC value of 8 µg/mL that is equal to the +ve control fluconazole. Compared to previously reported transition metal complexes bearing the same fluorinated counter anion but different type of ligand [42], it is clear that the presence of the pyridine ligand positively enhanced the activity of these prepared complexes **1**–**6**. This is could be attributed to the greater availability of the active sites of the metal centers in complexes **1**–**6**, that would make it easier for the metal to attack the cell walls of the different strains of bacteria, compared to their chelated analogous.

The pyridine, owing to its Lewis basic character rooted in its nitrogen lone pair, qualifies as the ligand for transition metals and is able to form metal complexes across the metals in the periodic table. It is usually a monodentate ligand having the capability to bind to metals in different proportions to produce a range of metal complexes. The electron-donating substituents at two and four positions help “N” in forming a stronger coordinate bond and enhance the stability of resultant complexes. Beside the chelating pyridine ligands, they provide appreciably higher stability compared to the monodentate pyridine moiety [58].

It is apparent from the results obtained that the nature of the coordinating ligands enhances the activity which can be explained on the basis of chelation theory [59]. Chelation increases the lipophilic nature of these complexes and this is likely to be responsible for a number of specific interactions with selected microorganisms, which enhance the complexes’ penetration into the lipid membrane of the microorganism cell wall, and therefore increases the activity of the complexes and resists further growth of the organism. The activity observed against the Gram-positive bacteria can be explained by considering the effect on lipopolysaccharide (LPS), the main component of the surface of Gram-positive bacteria [60]. LPS also is important in determining the virulence of Gram-negative pathogens and the outer membrane barrier function. These complexes can penetrate the bacterial cell membrane by coordination of the metal ion through nitrogen atoms to LPS which leads to the vandalism of the outer cell membrane and as a result inhibits growth of the bacteria.

## 3. Experimental Part

### 3.1. General

Unless otherwise stated, all chemicals and solvents are used as received from Sigma/Aldrich (Taukfirchen, Germany). Ag[B(C_6_F_5_)_4_] was synthesized according to the literature [34]. ^1^H and ^11^B NMR measurements were performed on an BRUKER 400 MHZ spectrometer (Bruker Optics, Ettlingen, Germany), using deuterated dimethylsulfoxide (DMSO-*d*_6_) as a solvent. Bruker alpha spectrometer was used to record the FT-IR spectra (Bruker Optics, Ettlingen, Germany) in the region 4000–400 cm^−1^ using KBr pellets. JEOL JES-FA 200 spectrometer was used for EPR spectra determination. At a microwave frequency of 9.27 GHz and a power of 5mW, the spectra were recorded with samples concentration of 10^−4^ mol/L in dried DMSO. Thermal analyses were performed using a PCT-2 A Thermo Balance analyzer (ThermoFisher Scientific, Waltham, MA, USA) at a heating rate of 10 °C/min in the range of 25 to 800 °C under nitrogen atmosphere. UV–Visible spectra were recorded in a solution with concentration of 10^−4^ M at 298 K using a UV-2401PC UV–Visible spectrophotometer (Shimadzu Corporation, Kyoto, Japan). Elemental analyses were carried out using a Vario EL metal analyzer (Elementar, Langenselbold, Germany).

### 3.2. Synthesis of Complexes **1**–**6**

Ag[B(C_6_F_5_)_4_] (1.00 g, 1.27 mmol) was dissolved in (25 mL) of dry acetonitrile, and specific amount (0.64 mmol) of anhydrous MX_2_ (M = Mn, Fe, Co, Ni, Cu, Zn, and X = Cl, Br) was added to the solution. The resulting mixture was stirred overnight in the dark. The precipitate (AgX) was removed and the filtrate was dried under reduced pressure, the resulting powder was dissolved in enough amount of dry dichloromethane, then an excess amount of dry pyridine was added to the solution and left for 30 min with continuous stirring. Then, the solution was stripped under reduced pressure. The resulting solid was washed with Et_2_O and hexane to yield the analytically pure product.

### 3.3. Computational Method

All density functional theory (DFT) calculations were performed using Gaussian 16 software [60]. The molecular geometries of the [MPy_6_]^2+^ complexes (M = Mn, Fe, Co, Ni, Cu, Zn) were fully optimized using the ωB97XD functional [61,62], which incorporates empirical dispersion corrections. The LANL2DZ pseudopotential basis set was employed for transition metal atoms to account for relativistic effects, while the 6-31+G(d) basis set was used for non-metal atoms (C, N, H). Frequency calculations at the same level of theory confirmed the absence of imaginary vibrational modes, ensuring that the optimized structures correspond to true energy minima. Vibrational frequencies were unscaled and directly compared to experimental IR spectra. The IR spectra were derived from the computed vibrational frequencies, with peak intensities calculated using the harmonic approximation.

### 3.4. Antimicrobial Properties

The clinical isolates used in this study were received from Ministry of Health-Jordan. The antimicrobial activity of the complexes was measured using agar diffusion and micro-broth dilution minimum inhibition concentration methods, as reported previously [63].

## 4. Conclusions

Six novel metal(II) complexes of the general formula [MPy_6_][B(C_6_F_5_)_4_]_2_ (M = Mn, Fe, Co, Ni, Cu, Zn) were synthesized and characterized by elemental analysis and various spectroscopic techniques. The complexes exhibited broad-spectrum antibacterial activity against several bacterial strains. Among them, complex **6** demonstrated the highest antibacterial efficacy, while complexes **1**, **2**, and **4** displayed notable activity against *Shigella flexneri*, *Klebsiella pneumoniae*, and *Streptococcus pyogenes*, respectively. In contrast, complexes **3**, **5**, and **6** showed the lowest activity against *Pseudomonas aeruginosa*, *Escherichia coli*, and *Klebsiella pneumoniae*. Complexes **5** and **6** also exhibited remarkable antifungal activity against *Candida albicans*, with MIC values equivalent to the standard antifungal agent fluconazole. Density Functional Theory (DFT) calculations confirmed an overall octahedral geometry for all complexes, with tetragonal distortions observed in complexes **3**, **4**, and **5**.

## Figures and Tables

**Table 1:** Major FT-IR bands for complexes **1**–**6** (cm^−1^).

Complexes	ν(Ar-(C-F))			ν(C-H)	ν(C=C)	ν(C=N)
**1**	1368	1273	1083	2924	1597	1643
**2**	1369	1274	1084	3062	1597	1643
**3**	1369	1273	1085	3037	1597	1644
**4**	1369	1273	1084	3036	1596	1642
**5**	1369	1273	1084	3039	1597	1644
**6**	1370	1273	1085	3037	1596	1644

**Table 2:** Yield and analytical data for all complexes.

Complexes	N (%) Found (calc.)	H (%) Found (calc.)	C (%) Found (calc.)	F (%) Found (calc.)	Yields
**1** C_78_H_30_B_2_F_40_N_6_Mn (1887.15 g/mol)	4.49	1.57	49.58	40.22	88.9% 0.802 g
	(4.45)	(1.60)	(49.63)	(40.26)	
**2** C_78_H_30_B_2_F_40_N_6_Fe (1888.14 g/mol)	4.46	1.61	49.66	40.27	86.6% 0.781 g
	(4.45)	(1.60)	(49.61)	(40.24)	
**3** C_78_H_30_B_2_F_40_N_6_Co (1891.14 g/mol)	4.48	1.59	49.47	40.10	98.0% 0.886 g
	(4.44)	(1.60)	(49.53)	(40.17)	
**4** C_78_H_30_B_2_F_40_N_6_Ni (1890.14 g/mol)	4.41	1.67	49.46	40.13	71.4% 0.646 g
	(4.44)	(1.60)	(49.53)	(40.18)	
**5** C_78_H_30_B_2_F_40_N_6_Cu (1895.15 g/mol)	4.40	1.62	49.43	39.97	94.9% 0.860 g
	(4.43)	(1.59)	(49.41)	(40.08)	
**6** C_78_H_30_B_2_F_40_N_6_Zn (1898.06 g/mol)	4.36	1.61	49.41	40.15	60.9% 0.552 g
	(4.43)	(1.59)	(49.36)	(40.04)	

**Table 3:** Thermal analysis data for all complexes.

Complexes	Stage	Temp. Range (°C)	Mass Loss %	Total Mass Loss %
**1**	1	94–196	16.88	94.96
	2	196–281	43.10	
	3	281–366	19.07	
**2**	1	34–116	16.85	92.77
	2	116–165	8.38	
	3	165–261	15.21	
	4	261–287	37.59	
**3**	1	49–81	4.66	94.82
	2	81–114	4.67	
	3	114–227	16.88	
	4	227–321	54.26	
**4**	1	37–116	8.40	96.17
	2	116–205	12.76	
	3	205–278	52.85	
	4	278–392	12.20	
**5**	1	36–130	8.55	90.59
	2	130–189	12.63	
	3	189–214	20.18	
	4	214–247	7.87	
	5	247–296	35.07	
**6**	1	41–151	8.47	99.36
	2	151–164	16.66	
	3	164–327	59.02	

**Table 4:** UV–Vis data for complexes **1**–**6**.

Complexes	Wavelength [nm]	Absorptivity	Transitions
**1**	316.0	1.340	π→π^*^
	364.0	0.578	π→π^*^
	416.0	0.253	n→π^*^
**2**	315.0	0.397	π→π^*^
	388.5	0.427	π→π^*^
	426.5	0.360	π→π^*^
	468.0	0.396	n→π^*^
**3**	275.5	3.084	π→π^*^
	571.0	0.249	n→π^*^
**4**	273.0	5.000	π→π^*^
	321.5	0.289	n→π^*^
**5**	272.0	2.608	π→π^*^
	309.5	0.215	π→π^*^
	322.5	0.195	n→π^*^
**6**	273.5	2.421	π→π^*^
	356.5	0.079	π→π^*^
	397.5	0.067	n→π^*^

**Table 5:** Agar well diffusion (mm) of all complexes against a number of bacteria after 24 h.

Tested Compounds	Gram (−) Bacteria						Gram (+) Bacteria	
	*Ec*	*Pm*	*Sf*	*Kp*	*Pa*	*Cf*	*Sa*	*Sp*
**1**	21.3	19.1	24	15.9	14.2	21.9	17.3	20.6
**2**	13	21.8	18.8	25.1	13.5	19.4	17.6	18.5
**3**	12.7	19.7	17.8	14.9	9.7	15.2	21.9	21.3
**4**	11.2	20.6	16.4	18.3	16.2	17	20.9	25.3
**5**	8.9	16.2	14.7	13.3	12.5	17.8	21.2	23.1
**6**	13.3	22.1	20.7	8.9	20.5	20	20.6	27.3
**DMSO** **(−ve control)**	Not active	Not active	Not active	Not active	Not active	Not active	Not active	Not active
**Oxytetracycline (+ve control)**	16	15	14	13	15	14	16	14

*Ec*: *Escherichia coli*, *Pm*: *Proteus mirabilis*, *Sf*: *Shigella flexneri*, *Kp*: *Klebsiella pneumoniae*, *Pa*: *Pseudomonas aeruginosa*, *Cf*: *Citrobacter freundii*, *Sa*: *Staphylococcus aureus*, *Sp*: *Streptococcus pyogenes*.

**Table 6:** Minimum inhibitory concentration (MIC, µg/mL) of all complexes against some clinical bacterial isolates using agar well diffusion after 24 h.

Tested Compounds	Gram (−) Bacteria						Gram (+) Bacteria		Fungi
	*Ec*	*Pm*	*Sf*	*Kp*	*Pa*	*Cf*	*Sa*	*Sp*	*Ca*
**1**	16	16	8	128	128	16	64	32	64
**2**	128	16	16	8	128	16	64	64	32
**3**	256	16	64	128	512	128	16	16	128
**4**	256	16	128	16	128	64	32	8	32
**5**	512	128	128	128	128	64	16	16	8
**6**	256	16	16	512	32	32	32	4	8
**DMSO** **(−ve control)**	Not active	Not active	Not active	Not active	Not active	Not active	Not active	Not active	Not active
**Oxytetracycline (+ve control)**	4	2	8	2	8	8	---	8	Not active
**Fluconazole** **(+ve control)**	Not active	Not active	Not active	Not active	Not active	Not active	Not active	Not active	8

## Data Availability

The Original contributions presented in this study are included in the article/Supplementary Material. Further inquiries can be directed to the corresponding author.

## Supplementary Materials

The following supporting information can be downloaded at: https://www.mdpi.com/article/10.3390/molecules30153121/s1, Figure S1: FT-IR spectrum of complex 1; Figure S2: FT-IR spectrum of complex 2; Figure S3: FT-IR spectrum of complex 3; Figure S4: FT-IR spectrum of complex 4; Figure S5: FT-IR spectrum of complex 6; Figure S6: 11B-NMR of complex 1; Figure S7: 11B-NMR of complex 2; Figure S8: 11B-NMR of complex 3; Figure S9: 11B-NMR of complex 5; Figure S10: 11B-NMR of complex 6; Figure S11: 1H-NMR of complex 6; Figure S12: TGA/DTG curves of complex 2; Figure S13: TGA/DTG curves of complex 3; Figure S14: TGA/DTG curves of complex 4; Figure S15: TGA/DTG curves of complex 5; Figure S16: TGA/DTG curves of complex 6; Figure S17: IR spectrum [Zn(Py)6]2+ complex; Figure S18: IR spectrum [Cu(Py)6]2+ complex; Figure S19: IR spectrum [Ni(Py)6]2+ complex; Figure S20: IR spectrum [Co(Py)6]2+ complex; Figure S21: IR spectrum [Fe(Py)6]2+ complex; Figure S22: IR spectrum [Mn(Py)6]2+ complex; Table S1: IR-active M–N stretching frequencies and corresponding bond lengths for [M(Py)6]2+ complexes (M = Zn, Cu, Ni, Co, Fe, Mn). Bond length values correspond to the DFT-optimized structures presented in Figure 1.
