# Supplementary material for: Anti-Bacterial and Anti-Fungal Properties of a Set of Transition Metal Complexes Bearing a Pyridine Moiety and [B(C6F5)4]2 as a Counter Anion
Source: Molecules. 2025 Jul 25;30(15):3121. doi: 10.3390/molecules30153121 (PMC12348494; doi:10.3390/molecules30153121)
Supplement: Supplementary file 1 [file molecules-30-03121-s001.zip › molecules-3766994-supplementary.pdf]

Anti-Bacterial and Anti-Fungal Properties of a Set of Transition Metal Complexes Bearing a Pyridine Moiety and $[B(C_6F_5)_4]_2$ as a Counter Anion

Ahmed K. Hijazi[1, 2*]; Mohammad El-khateeb[2]; Ziyad A. Taha[2]; Mohammed I. Alomari[1], Noor M. Khwaileh[2]; Abbas I. Alakhras[3]; Waleed M. Al-Momani[4]; Ali Elrashidi[5, 6]; Ahmad S. Barham[7]

[1] Department of Chemistry, College of Arts and Sciences, University of Petra, P.O. Box 961343, Amman 11196, Jordan. E-mail: ahmad.hijazi@uop.edu.jo

[2] Department of Chemical Sciences, Faculty of Science and Arts, Jordan University of Science and Technology, P.O. Box 3030, Irbid 22110, Jordan. E-mail: akhijazi@just.edu.jo

[3] Department of Chemistry, College of Science, Imam Mohammad Ibn Saud Islamic University, P.O. Box 5701, Riyadh 11432, Saudi Arabia

[4] Department of Basic Medical Sciences, Faculty of Medicine, Yarmouk University, Irbid, Jordan

[5] Electrical Engineering Department, College of Engineering, University of Business and Technology, Jeddah 23435, Saudi Arabia

[6] Engineering Mathematics Department, Faculty of Engineering, Alexandria University, Alexandria 21544, Egypt

[7] Department of Chemistry, School of Science, The University of Jordan, Amman 11942, Jordan

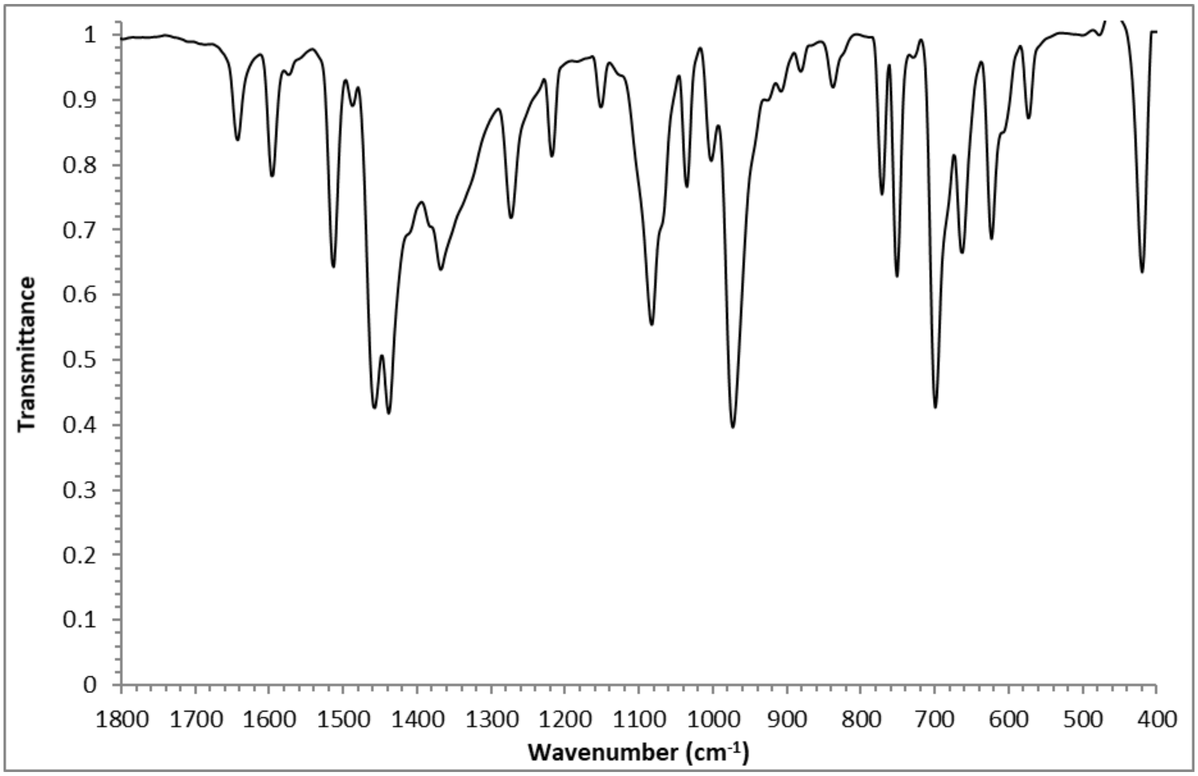

Figure S1: FT-IR spectrum of complex **1**

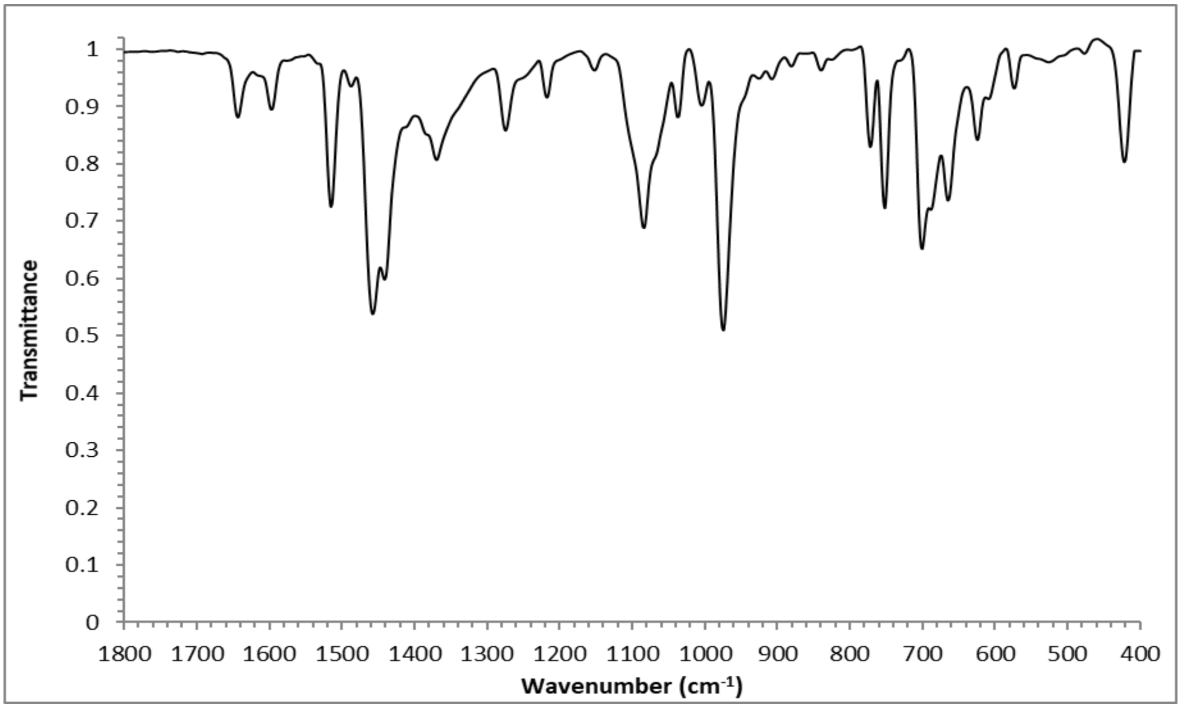

Figure S2: FT-IR spectrum of complex **2**

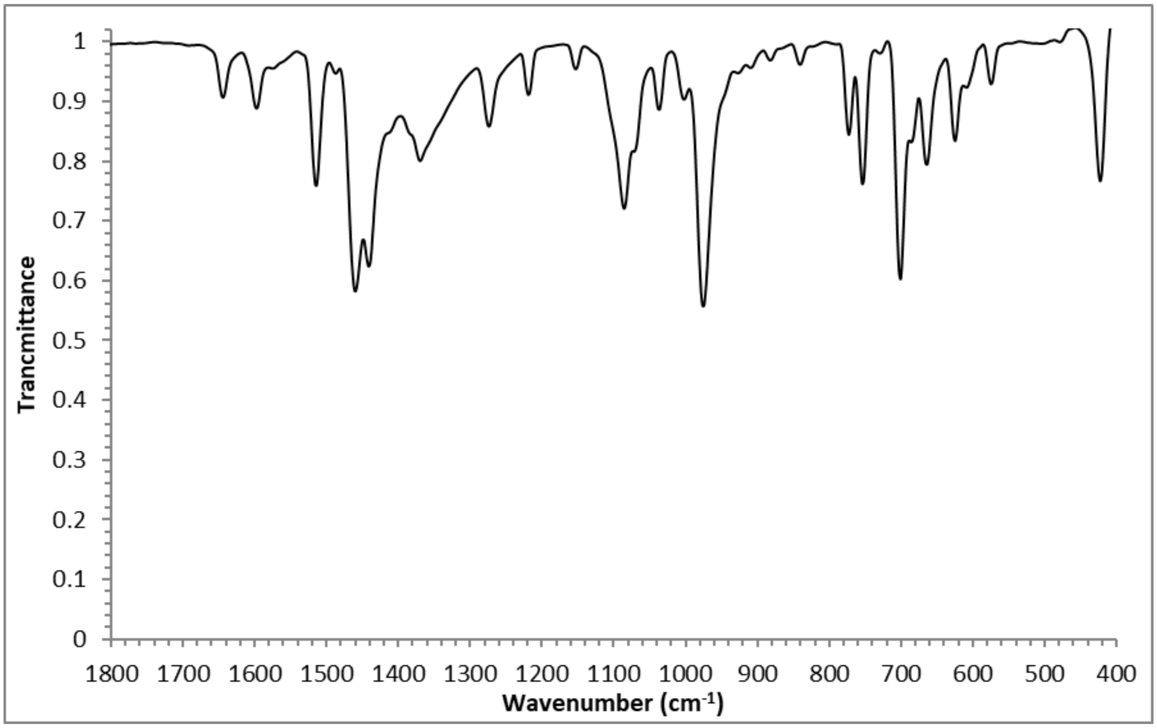

Figure S3: FT-IR spectrum of complex **3**

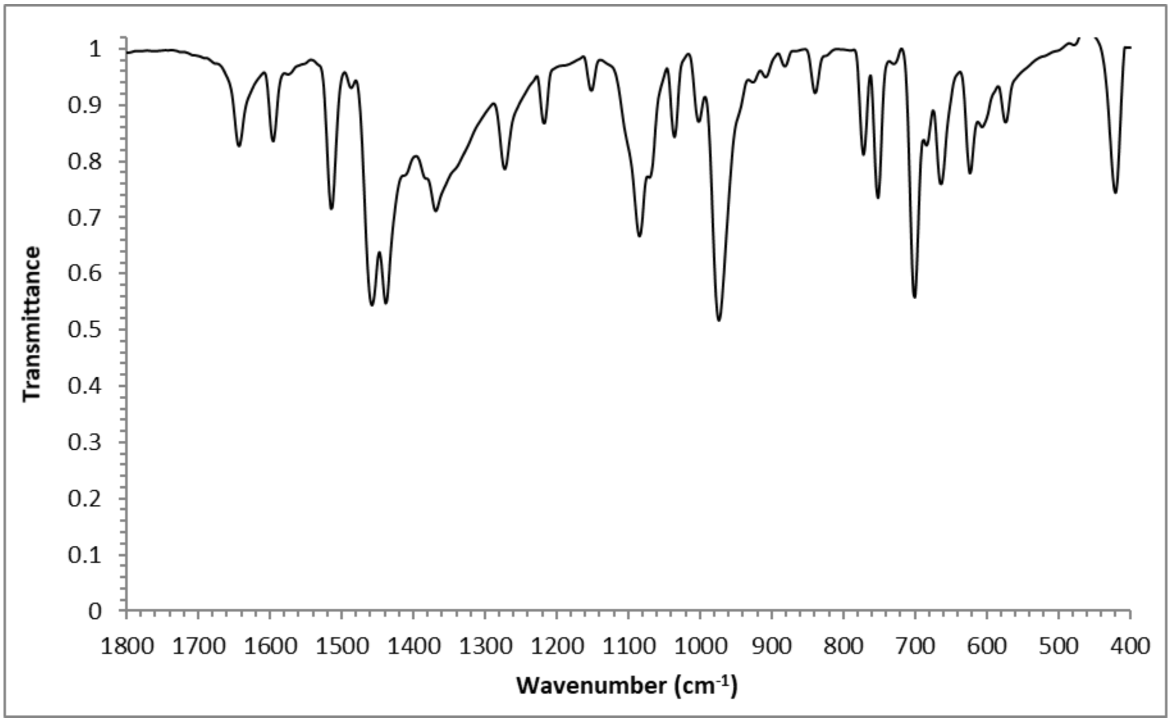

Figure S4: FT-IR spectrum of complex **4**

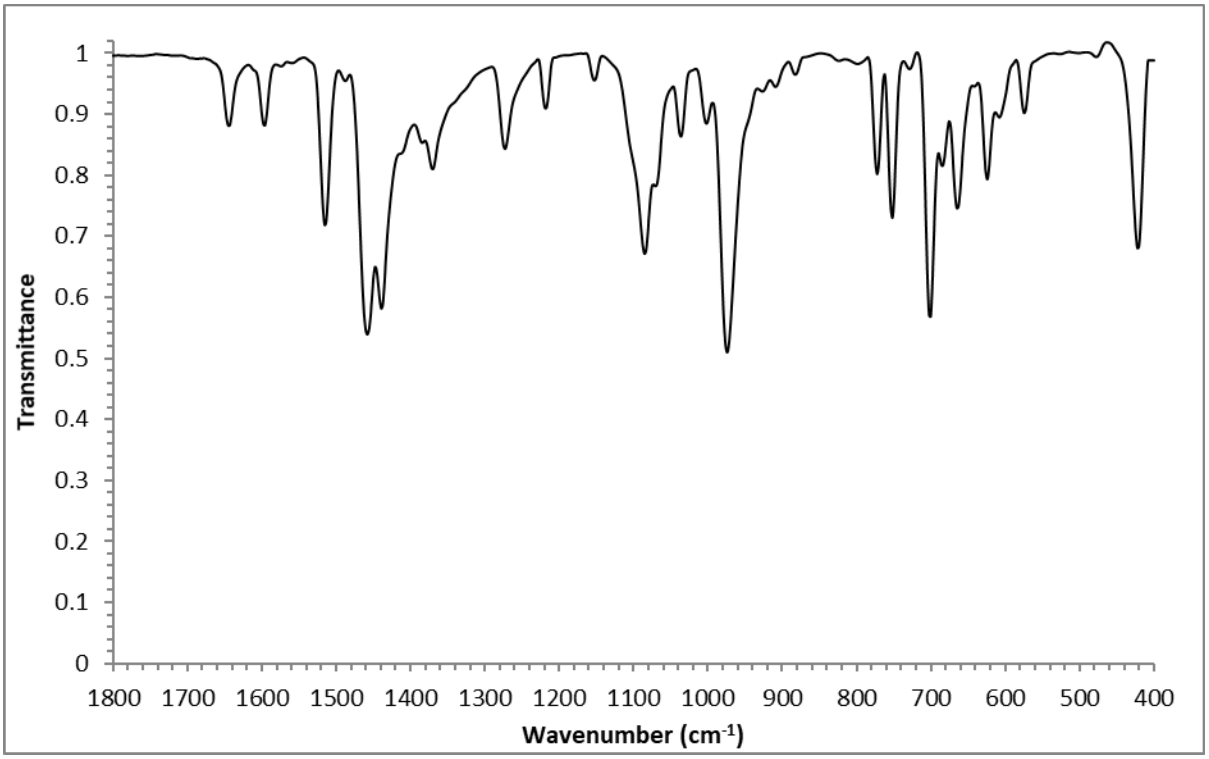

Figure S5: FT-IR spectrum of complex **6**

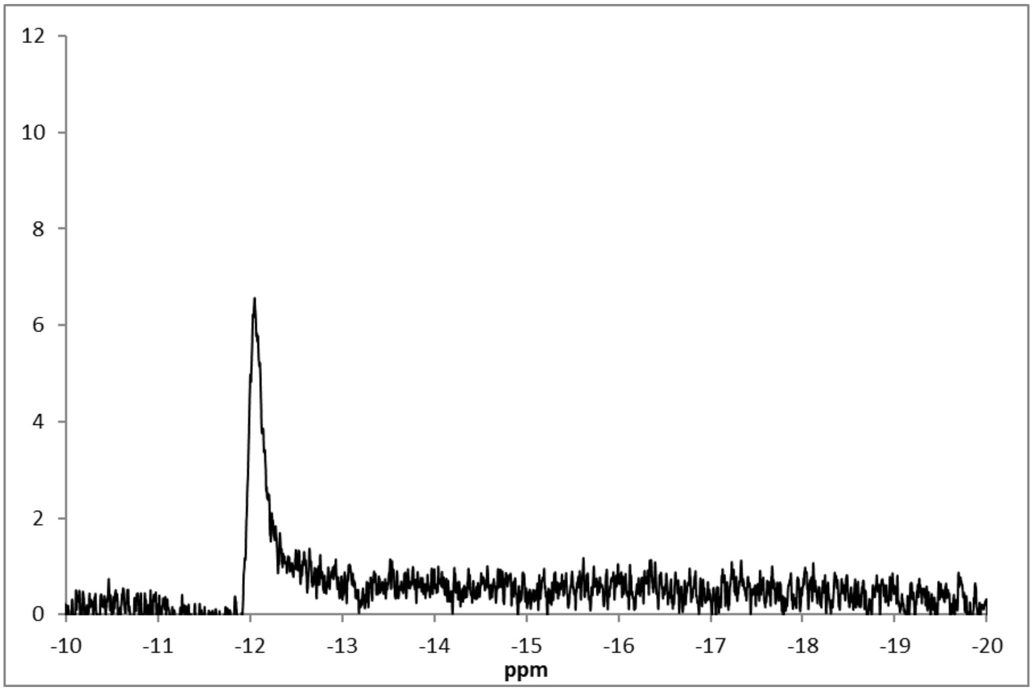

Figure S6: ^{11}B-NMR of complex **1**

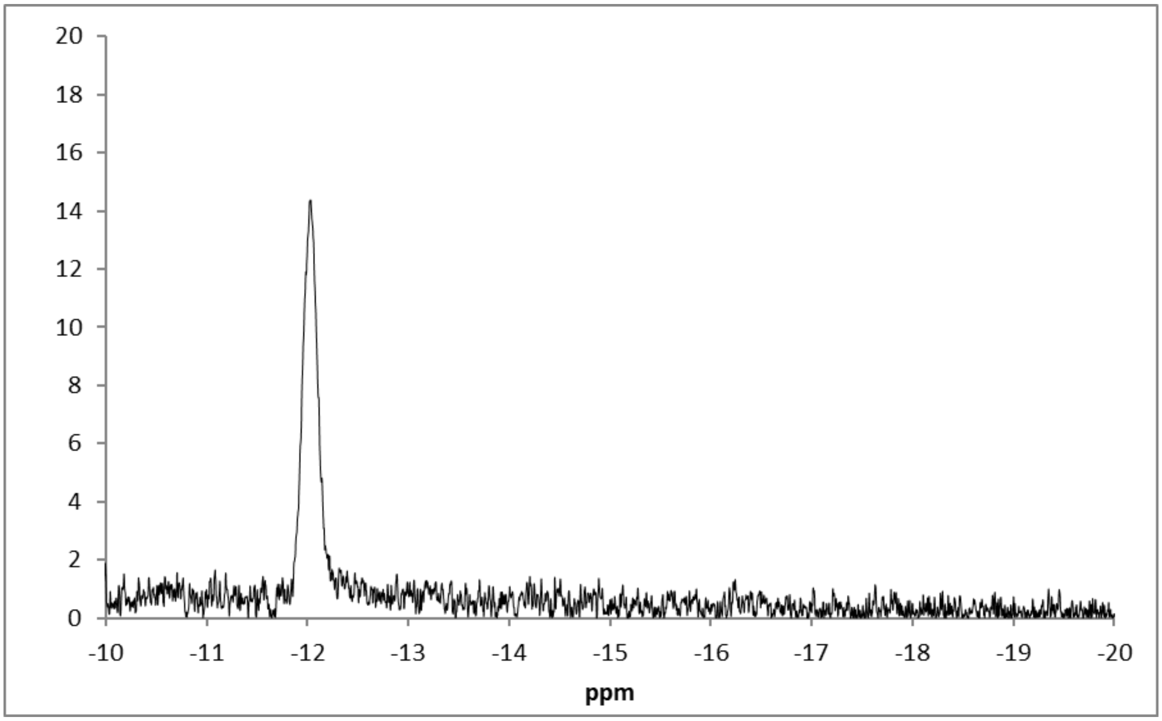

Figure S7: ^{11}B-NMR of complex **2**

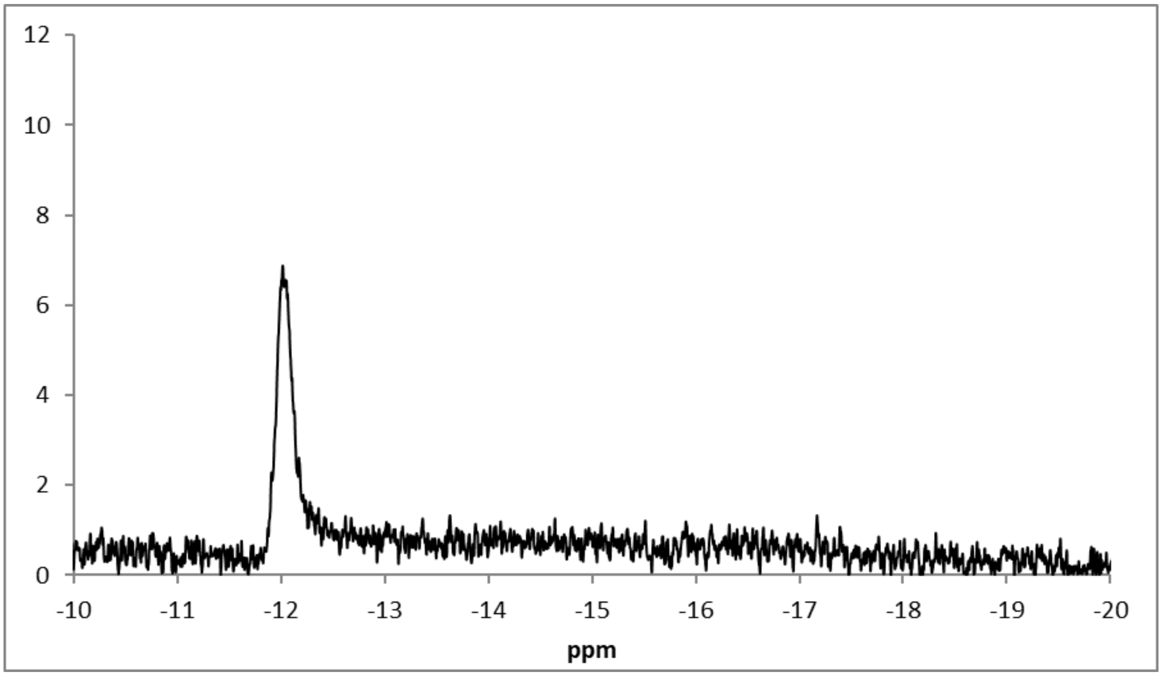

Figure S8: ^{11}B-NMR of complex **3**

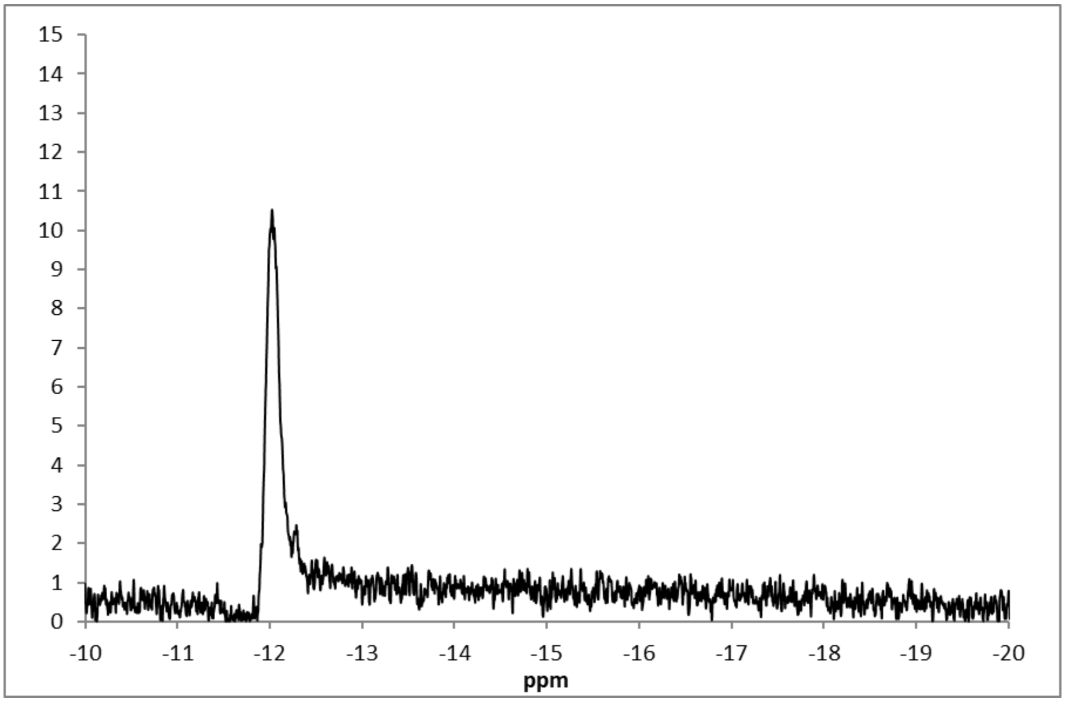

Figure S9: ^{11}B-NMR of complex **5**

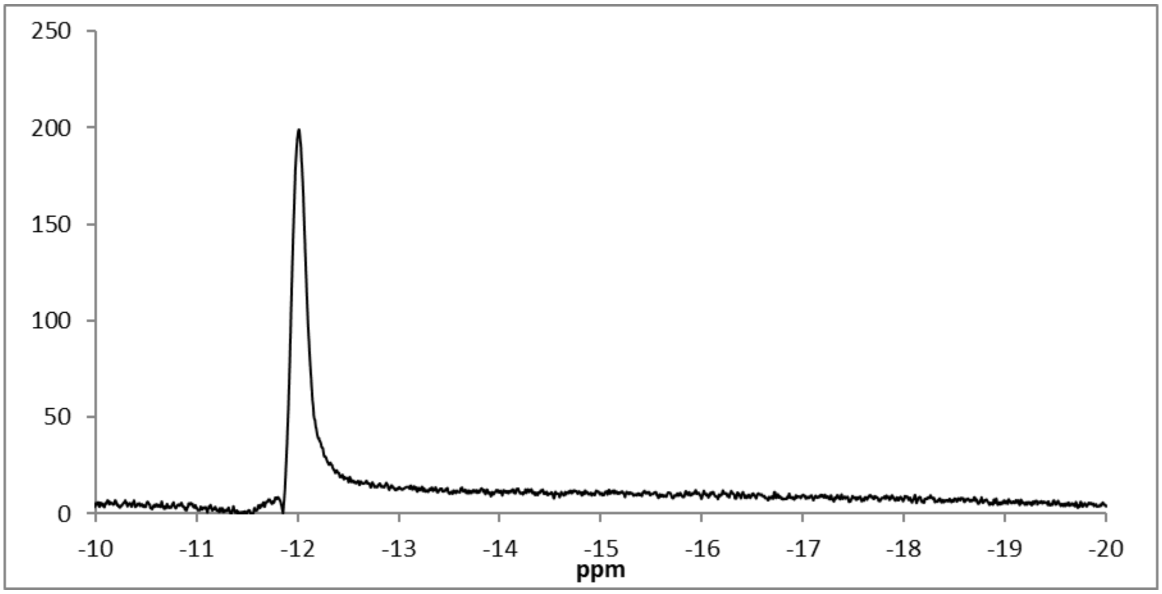

Figure S10: ^{11}B-NMR of complex **6**

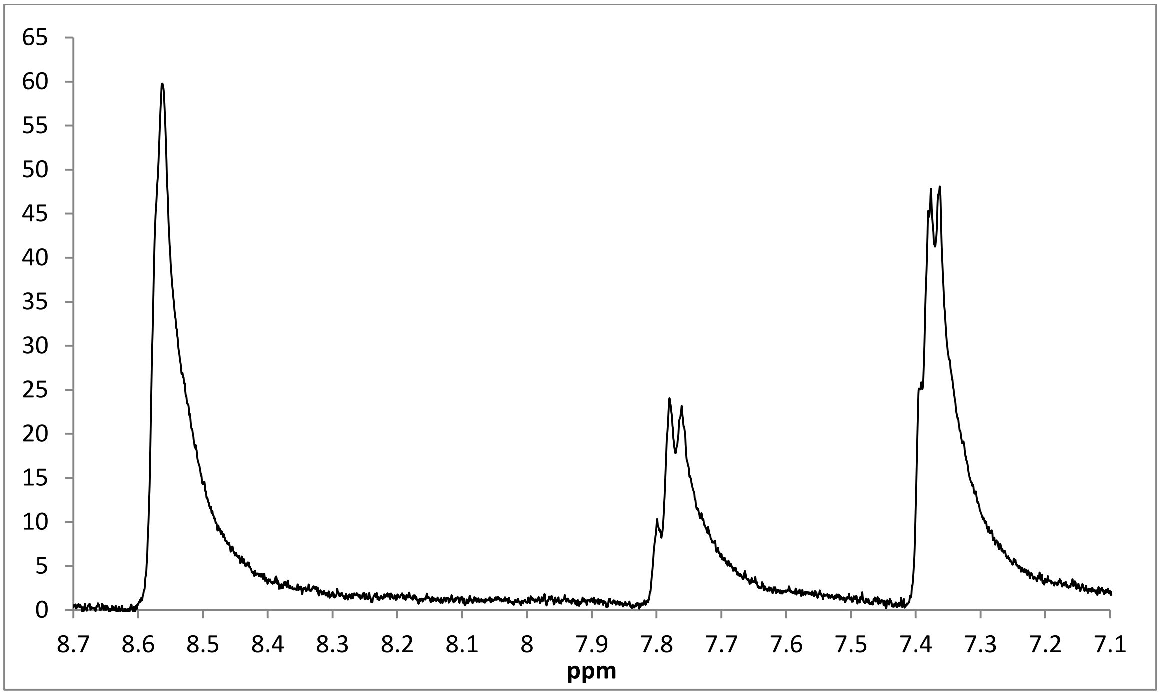

Figure S11: ^{1}H-NMR of complex **6**

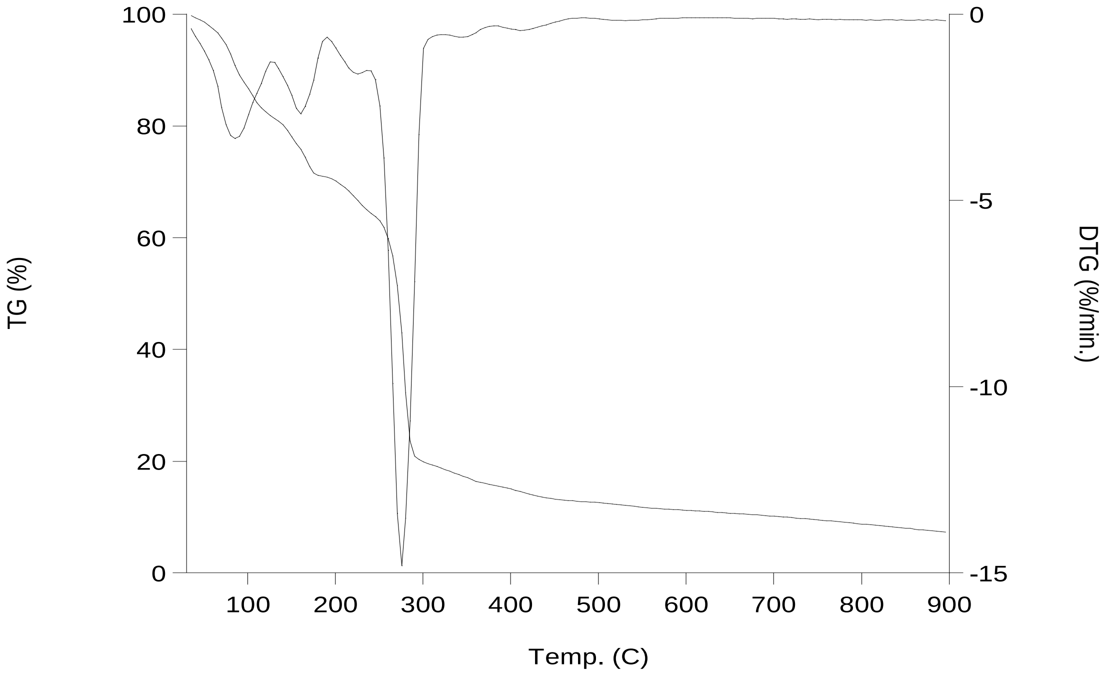

Figure S12: TGA/DTG curves of complex **2**

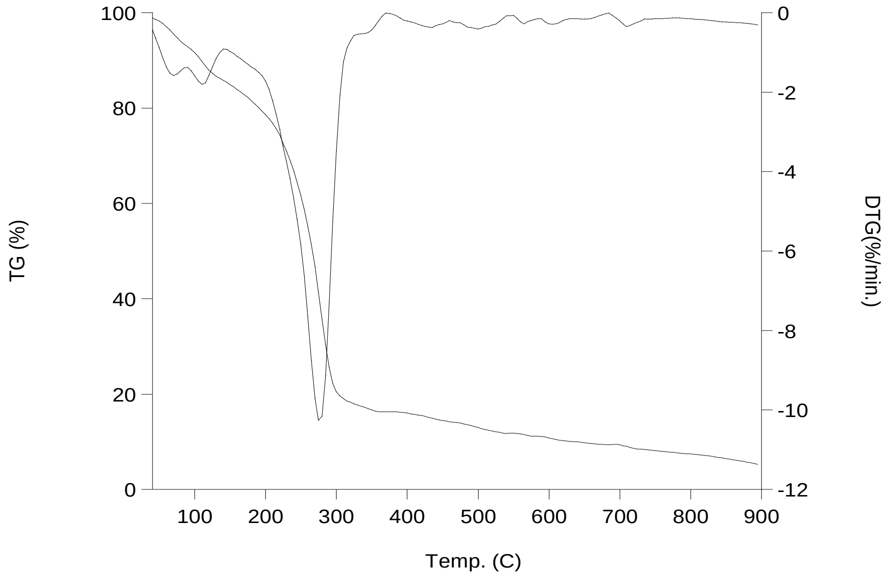

Figure S13: TGA/DTG curves of complex **3**

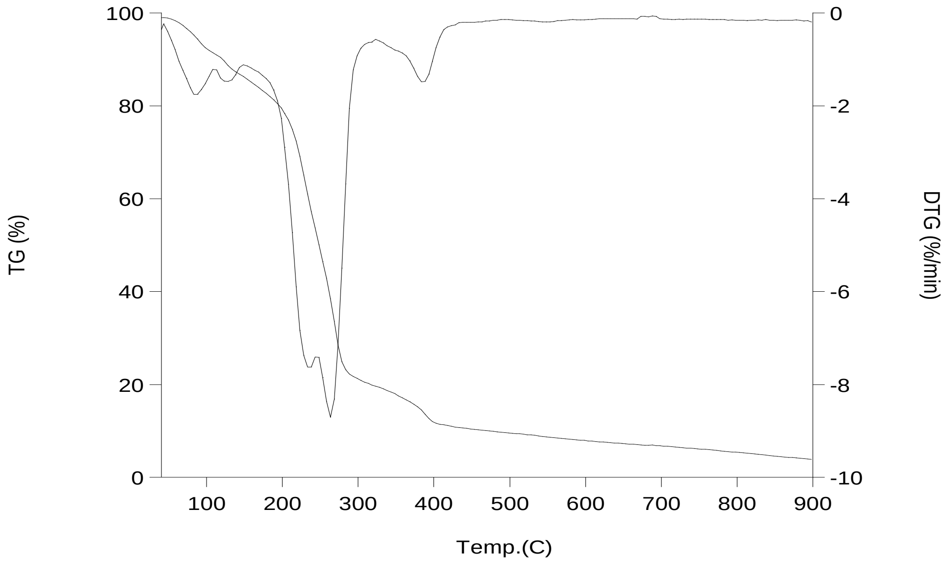

Figure 14: TGA/DTG curves of complex **4**

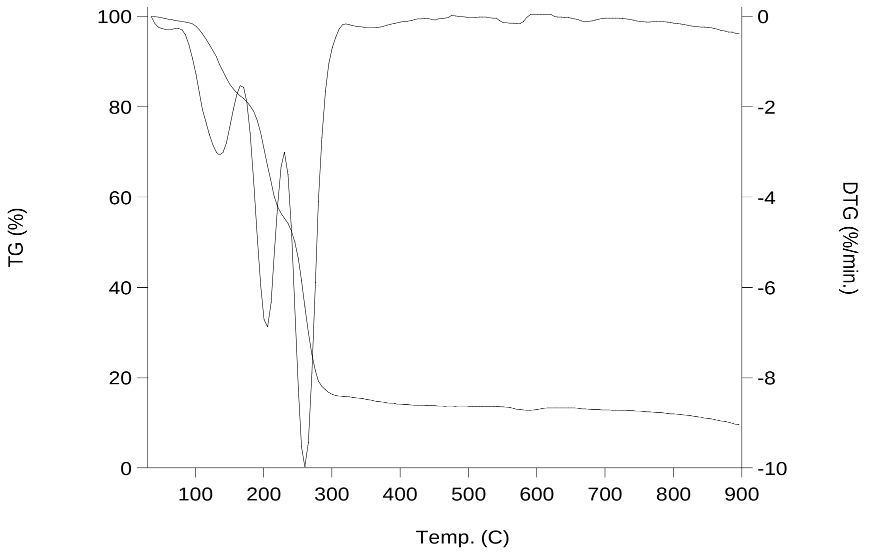

Figure S15: TGA/DTG curves of complex **5**

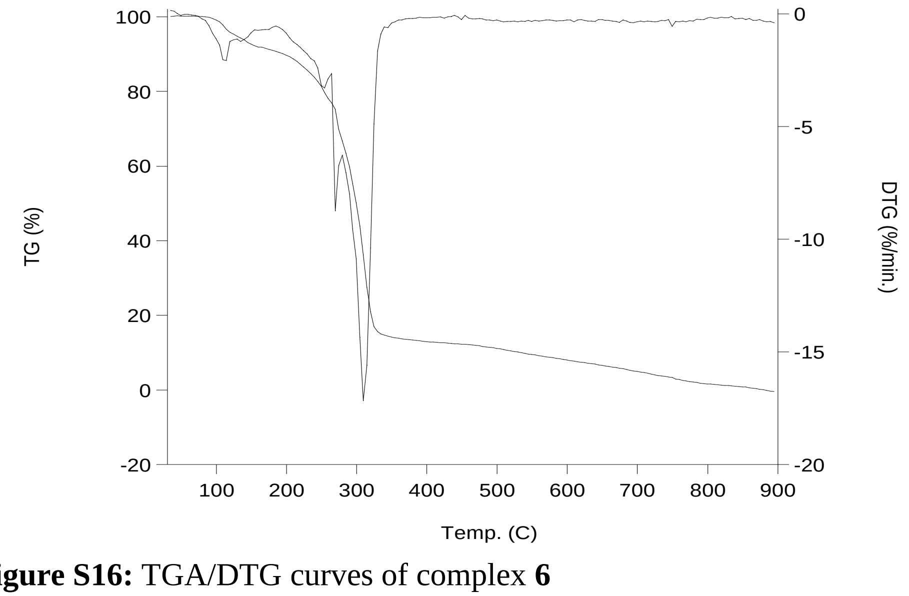

Figure S16: TGA/DTG curves of complex **6**

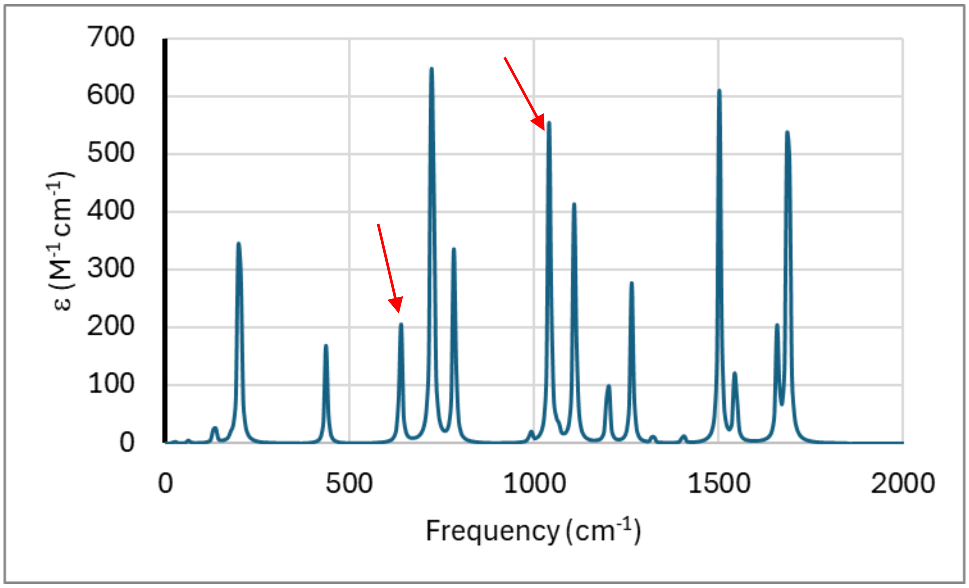

Figure S17: IR spectrum $[Zn(Py)_6]^{2+}$ complex

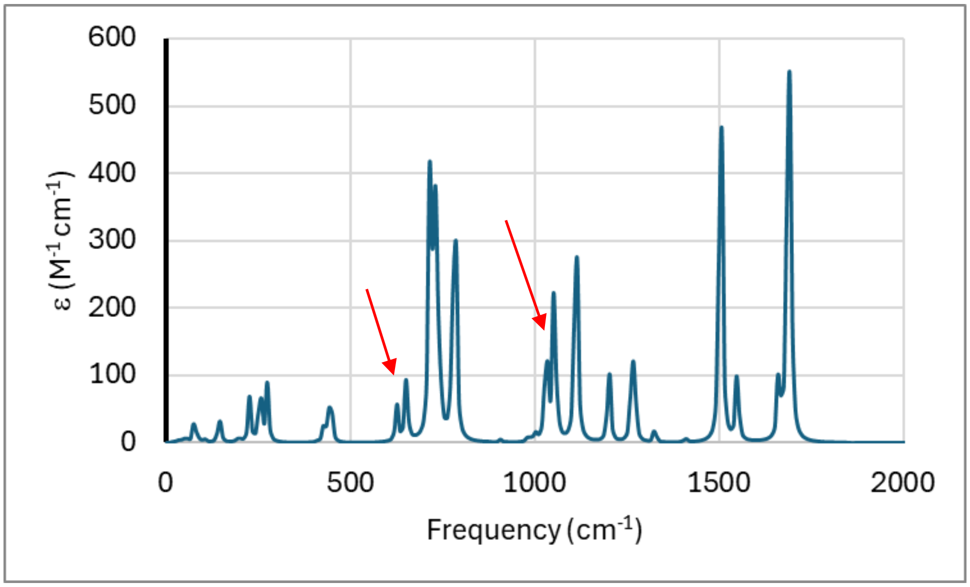

Figure S18: IR spectrum $[Cu(Py)_6]^{2+}$ complex

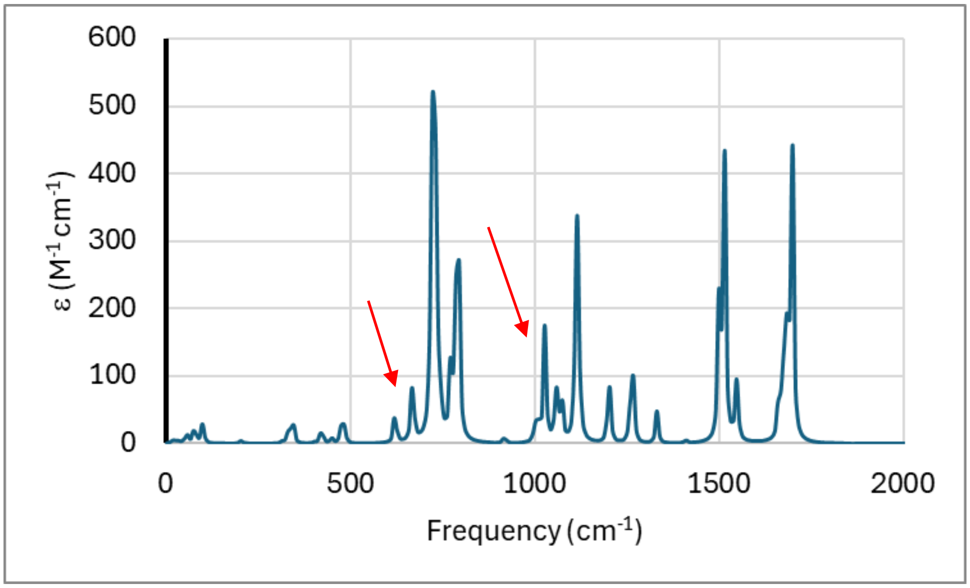

Figure S19: IR spectrum $[Ni(Py)_6]^{2+}$ complex

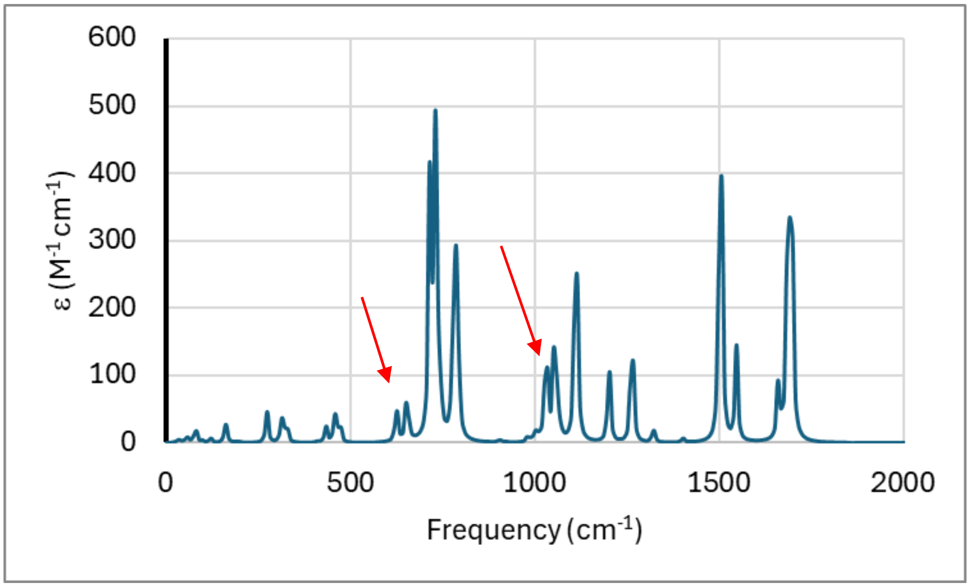

Figure S20: IR spectrum $[Co(Py)_6]^{2+}$ complex

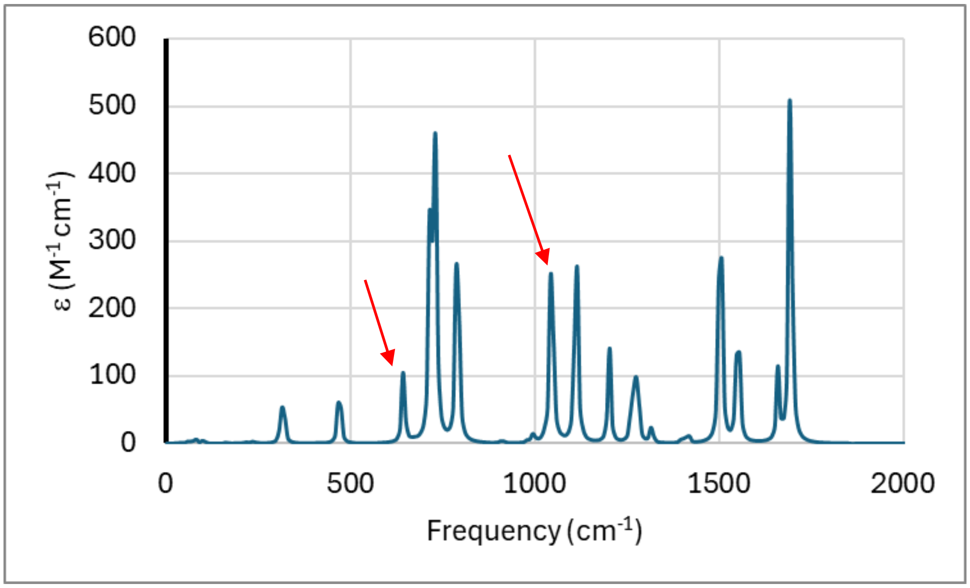

Figure S21: IR spectrum $[Fe(Py)_6]^{2+}$ complex

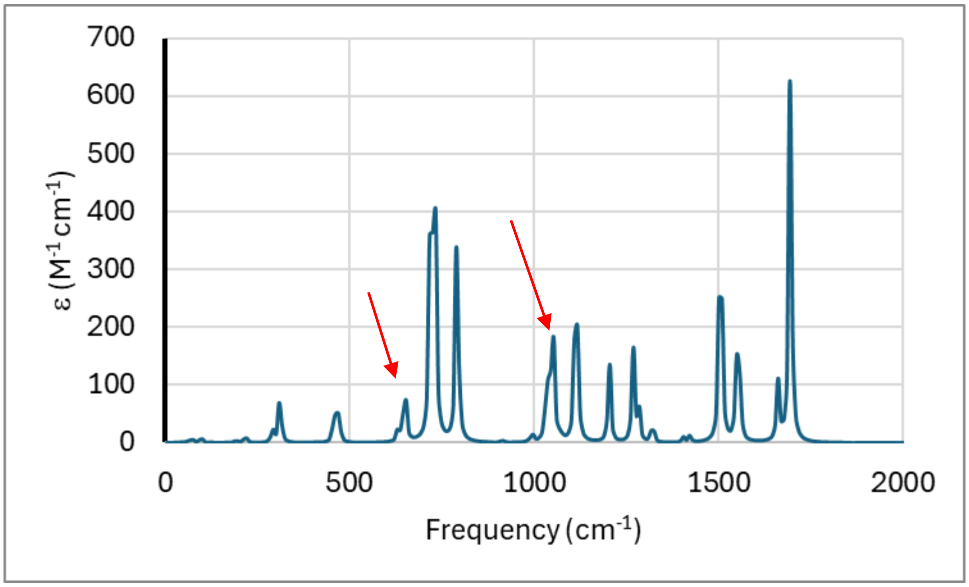

Figure S22: IR spectrum $[Mn(Py)6]^{2+}$ complex

Table S1: IR-active M–N stretching frequencies and corresponding bond lengths for $[M(Py)_6]^{2+}$ complexes (M = Zn, Cu, Ni, Co, Fe, Mn). Bond length values correspond to the DFT-optimized structures presented in Figure 1.

	Zn(II) complex		Cu(II) complex		Ni(II) complex	
	Wavenumber (cm^{-1})	M-N bond length (Å)	Wavenumber (cm^{-1})	M-N bond length (Å)	Wavenumber (cm^{-1})	M-N bond length (Å)
1	640.03	2.29	626.98	2.62	622.47	3.36
2	640.03	2.29	651.43	2.1	669.95	1.93
3	640.04	2.29	654.2	2.09	670.13	1.93
4	1043.38	2.29	1032.21	2.62	1028.33	3.36
5	1043.38	2.29	1051.94	2.1	1073	1.93
6	1043.38	2.29	1055.51	2.09	1073.37	1.93

	Co(II) complex		Fe(II) complex		Mn(II) complex	
	Wavenumber (cm^{-1})	M-N bond length (Å)	Wavenumber (cm^{-1})	M-N bond length (Å)	Wavenumber (cm^{-1})	M-N bond length (Å)
1	625.88	2.55	643.15	2.16	630.78	2.21
2	654.49	2.03	645.39	2.13	645.46	2.16
3	655.2	2.03	646.13	2.12	650.76	2.14
4	1033.01	2.55	1044.8	2.16	1033.74	2.21
5	1054.24	2.03	1047.29	2.13	1048.03	2.16
6	1056.2	2.03	1048.5	2.12	1049.53	2.14
